# Partitioning β-diversity reveals that invasions and extinctions promote the biotic homogenization of Chilean freshwater fish fauna

**DOI:** 10.1371/journal.pone.0238767

**Published:** 2020-09-08

**Authors:** Sergio A. Castro, Pablo Rojas, Irma Vila, Evelyn Habit, Jaime Pizarro-Konczak, Sebastián Abades, Fabián M. Jaksic

**Affiliations:** 1 Laboratorio de Ecología y Biodiversidad, Departamento de Biología, Facultad de Química y Biología, Universidad de Santiago de Chile, Santiago, Chile; 2 Center of Applied Ecology and Sustainability, Pontificia Universidad Católica de Chile, Santiago, Chile; 3 Laboratorio de Limnología, Departamento de Ciencias Ecológicas, Universidad de Chile, Santiago, Chile; 4 Departamento de Sistemas Acuáticos, Facultad de Ciencias Ambientales y Centro de Ciencias Ambientales EULA, Universidad de Concepción, Concepción, Chile; 5 Departamento de Ingeniería Geográfica, Universidad de Santiago de Chile, Santiago, Chile; 6 Centro de Genómica y Bioinformática, Facultad de Ciencias, Universidad Mayor, Santiago, Chile; University of Molise, Isernia, ITALY

## Abstract

**Aim:**

Exotic species’ introductions together with extinction of native species represent the main mechanisms driving biotic homogenization of freshwater fish assemblages around the world. While generally ichtyofaunistic realms transit towards biotic homogenization, for conservation purposes it is essential to understand what specific mechanisms are promoting it on particular areas or regions. Here, we report the occurrence of biotic homogenization in 29 Chilean watersheds, analyzing its β-diversity (including turnover and nestedness) and predicting future trends.

**Location:**

Continental Chile (18^o^–56^o^ S).

**Methods:**

We determined fish composition per basin for historical and current assemblages; extant native, exotic, and extinct species were recorded as 1 (presence) or 0 (absence) in two matrices basins × species. For each matrix, we calculated the turnover (β_sim_), nestedness (β_nes_), and β-diversity (β_sor_); then, we obtained Δβ_sim_, Δβ_nes_, and Δβ_sor_, as the arithmetical difference between basin pairs over time. In addition, we search for explanatory variables correlating Δβ_sim_, Δβ_nes_, and Δβ_sor_ with geographical and land use variables. Finally, simulating events of species introduction (i.e., invasion) and extinction, we generated 15 hypothetical assemblages, looking to establish future trends towards biotic change in Chilean basins.

**Results:**

Species turnover and β-diversity significantly decreased from historical to current assemblages (Δβ_sim_ = -0.084; Δβ_sor_ = -0.061, respectively), while the species nestedness did not show significant changes (Δβ_nes_ = 0.08). Biotic changes have been driven mainly by the introduction of 28 exotic species, with a minor role of extinctions (one species) and translocations (0 species) of native species. Changes in β-diversity were negatively correlated with area, elevation, and geographical distance between basins but not with land-use nor human population. Finally, the analysis of 15 future assemblages predicts a significant decrease of β-diversity and turnover, and an increase for species nestedness, this time promoted by an increase in the extinction of native species.

**Main conclusion:**

Chilean basins show a significant decrease of the distributional β-diversity and species turnover of the freshwater fish fauna, evidencing a trend towards biotic homogenization. This trend is shared with other Neotropical basins; however, specific mechanisms driving it show different magnitude. Changes in the β-diversity components do not show correlation with variables associated to land use, thus suggesting that casual introductions of freshwater fishes in Chile follow an opportunistic mode related to commercial use. According to future scenarios simulated, biotic homogenization should increase further, mainly as consequence of increased native extinctions.

## Introduction

Freshwater fish assemblages show a particular vulnerability to human activity and agents of global change [1]. These assemblages are characterized by a moderate or high species richness and endemism [2], have experimented a rise in their extinction rates that is concomitant with an increased invasion tide of exotic species [3, 4]. As result, the exotic fishes (i.e., non-native species) replace native ones, thus increasing compositional similarity among freshwater assemblages [5]. This process, called ‘biotic homogenization’ [6], leads to the loss of geographical turnover and historical bio-distinction of these assemblages [7]. Two decades of homogenization studies have been an important component in the conservation agenda at the biogeographical scale [8, 9]. Theoretical as well as empirical advances in invasion biology are needed to establish conservation measures for current and future fish communities [10].

Although homogenization studies in freshwater fish assemblages are increasing, most part of them come from temperate latitudes of the Northern Hemisphere [e.g., 11–20], evidencing a remarkable geographical bias. Because the species diversity is differentially distributed between hemispheres, continents, and regions [2], it is desirable to know whether biotic homogenization occurs in areas beyond those most frequently reported [10, 21]. This, not only to reveal the global nature of the process, but also to characterize its mechanisms, modes, and future trends in different regions [22]. This knowledge could enable managers to anticipate or to revert–if possible–their ecological and evolutionary consequences [10, 22–24].

Freshwater fish assemblages show that biotic homogenization is driven mainly by introduction of exotic species, and to a lesser extent by extinction of native species [10, 21, 24, 25]. Nevertheless, due to growing habitat modification, climate change, and impact of non-native species, an increase in the extinction of native ones is expected [26–29]; likewise, also it is expected that some non-native species will continue expanding their distribution [30]. Therefore, deepening our understanding on the processes driving species composition towards homogenization is of utmost importance, because these changes could accelerate in the near future [22, 31], and a therefore a prediction framework is urgently required [29, 30].

Due to the presence of natural geographical barriers that coincide with its political limits, continental Chile is considered a biogeographic island inside South America, and therefore, an attractive study system [32]. On its western slope the Andean Range imposes a set of basins latitudinally arranged, most part of them discharging at the Pacific Ocean [33]. The Chilean freshwater fish fauna is composed by 45 native species [34]. This richness increases with latitude, from 1 to 18 species per basin up to 40° S, and then it decreases to 6 species per basin [34, 35]. In spite of this low species richness, this freshwater fish fauna has high endemism (80%) and including relict taxa, result of a complex biogeographical origin in South America [33–37]. Additionally, the Chilean basins contain 28 exotic species, naturalized in different watersheds [38, 39]. Studies have shown biotic homogenization at the ichthyogeographical level (i.e., the biogeographical regions recognized for the freshwater fish sauna in continental Chile), whereas varied patterns have been observed at the basin level. Indeed, Marr et al. [40] reported that basins located in central Chile are homogenizing with other Mediterranean regions of the world, whereas Castro et al. [41] and Vargas et al. [42] found an incipient homogenization trend among biogeographical regions within Chile. All of these studies have used metrics based on similarity/dissimilarity (Sorensen and Jaccard), that do not allow understanding the processes underlying biotic homogenization [43–47]. Analysis based on β-diversity are more adequate to evaluate biotic homogenization [43–45] because it is considered that changes in β-diversity respond to two different phenomena: species nestedness and turnover [43–45, 48, 49]. The nestedness corresponds to β-diversity’s component due to changes by gain or loss of species among samples [44–47], while the species turnover can be defined as the ‘true’ replacement of species among biotas [49], as result of environmental and geographical limitations that have been established over time [43, 50, 51]. Thus, the rationale of approaches based on β-diversity is that species widely distributed provide a decreased β-diversity among assemblages, evidencing ultimately, the role of turnover and nestedness as spatial processes underlying biotic homogenization. In this context, because the latitudinal arrangement of the Chilean basins, these constitute an attractive biogeographical model for studying the current and future paths of fish diversity in the Neotropical realm.

In the present article, we aim to establish whether Chilean basins exhibiting a poor but highly endemic freshwater fish fauna, are undergoing biotic homogenization. Unlike previous studies carried out in Chile, we propose an analytic framework based on β-diversity and its components, in order to examine homogenization and its future trends in Chile. Thus, we consider three situations: a) historical (also known as pre-European), b) current species (post-European) composition on basin assemblages, and c) a set of hypothetical assemblages which were generated simulating occurrence of new invasion and extinction events. We hypothesized that: (i) if biotic homogenization is occurring among Chilean basins, then the current β-diversity components (one of them or both) should be significantly lesser than those historically established; and (ii) if future spread/extinction events intensifies homogenization, then future β-diversity components (one of them or both) should be significantly lesser than those currently established.

## Material and methods

### The basins

**Fig 1:**
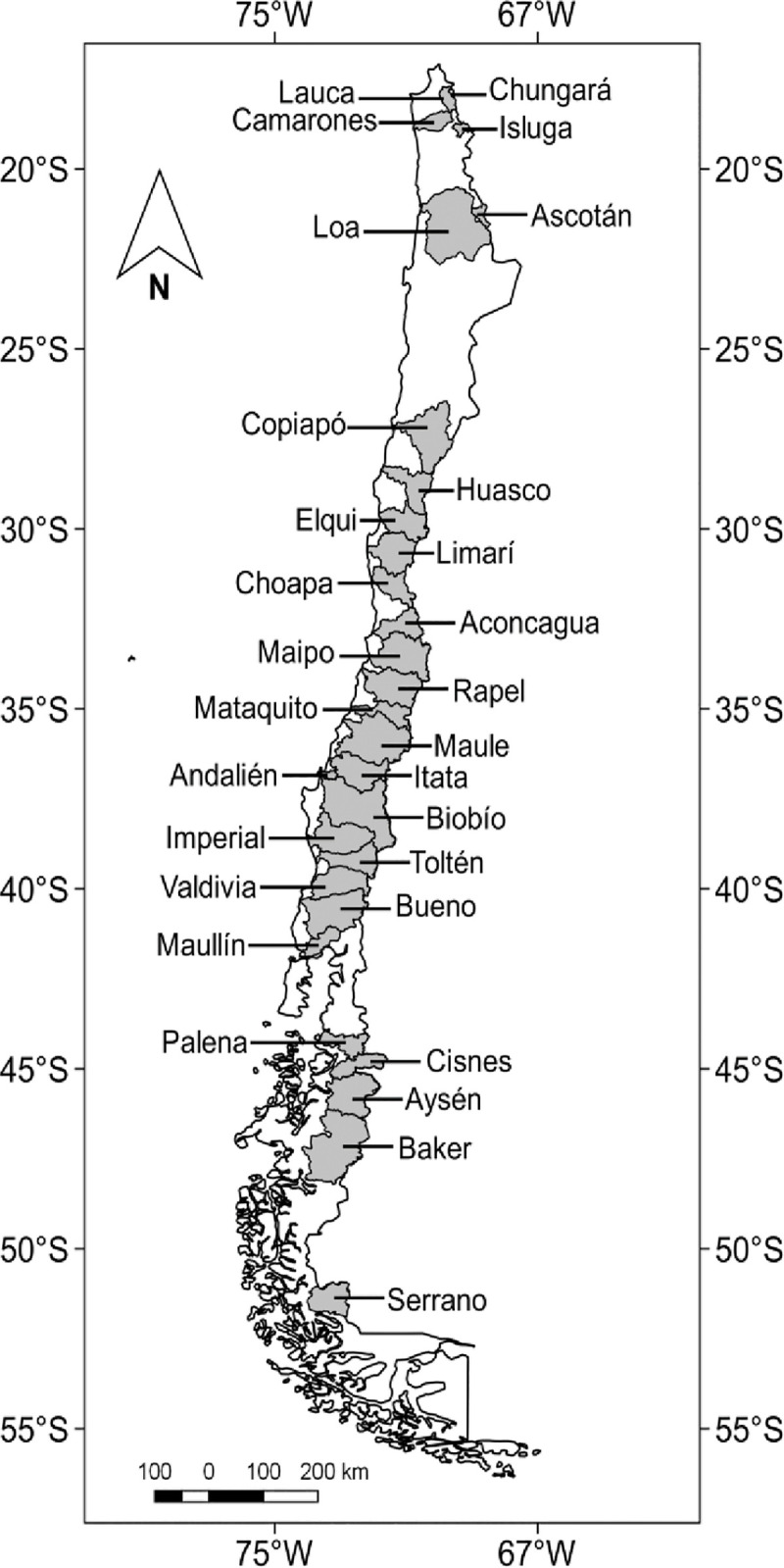
Geographic location of the 29 basins studied in continental Chile.

Continental Chile (17° 29’–56° 32’ S) has an extension of ca. 4,200 km, crossing a wide latitudinal gradient (Fig 1), with an approximate area of 755,776 km^2^. Within this territory, there are 101 major hydrographic basins [52], most of which correspond to permanent exorheic water courses (i.e., with superficial drainage heading outward) running from the Andean Ranges to the Pacific Ocean (i.e. from east to west; Fig 1). Exceptions are the basins as Ascotán, Chungará, Lauca, and Isluga (see Fig 1), which are located in the Andean highlands and whose regime is endorheic (i.e., with superficial drainage retained within the basin).

Currently, it is estimated that the composition of freshwater fish fauna has been satisfactorily documented for 29 basins [53, 54], all which were included in this study (Fig 1). Most native and all non-native species present in Chile are found on these 29 watersheds [54]. All of them are distributed along a latitudinal gradient that represents > 40% of the Chilean continental area (Fig 1) and encompasses the whole spectrum of ecoregions recognized for inland waters in Chile [33, 34, 53]. Although one of the basins is shared with Bolivia (Lauca), all data (environmental, geographic and biotic composition) refer to the Chilean moiety.

### Freshwater fish fauna

**Fig 2:**
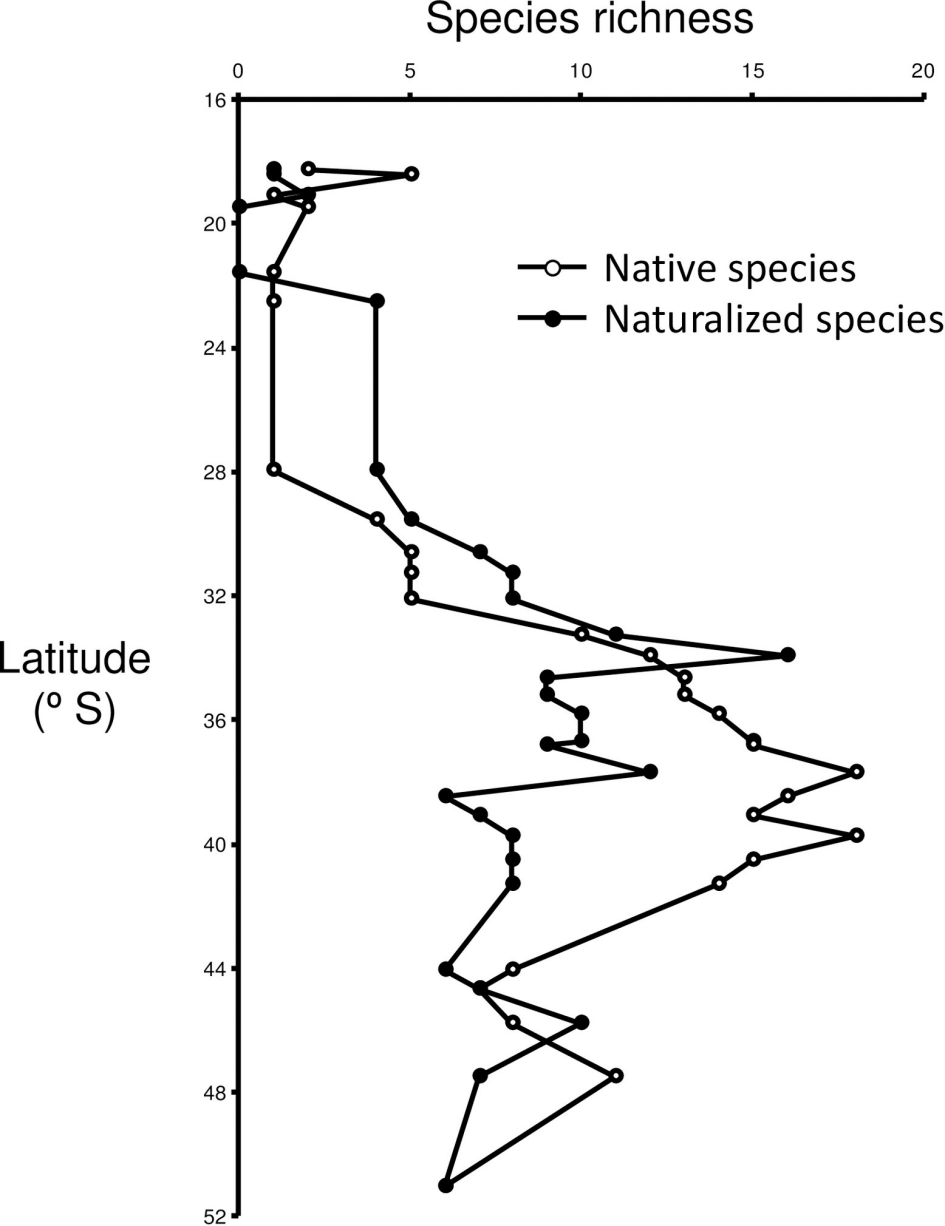
Native and naturalized species richness on Chilean basins according to latitudinal location.

Through a complete bibliographical review and authors’ personal records (Irma Vila and Evelyn Habit), we compiled a database with fish occurrence for each basin, distinguishing both native as exotic species. We labeled ‘native’ species as those that occur historically in a basin previous to the European colonization that started in mid-16^th^ century, and whose distribution is a result of eco-evolutionary processes in Chilean basins. In turn, ‘exotic’ species were those non-native species introduced since 1535, which currently show naturalized populations (i.e., established species) in a given Chilean basin. Additionally, we labeled as ‘translocated’ species those that while being native of some Chilean basin, were introduced into other Chilean watersheds where they did not historically occur [54]. Our review allowed us to exclude those species with strong marine or estuarine affinities or that may enter freshwater occasionally for only short time periods (see S1 File). In addition, the compositional information of each basin was based on cross-referencing information of the biota described for each one and the geographical distribution documented for freshwater fishes. In this review, we used as sources scientific publications and technical governmental reports. In particular, all these publications (93 titles) cover ca. 90 years, between 1927 and 2017 (S1 File). Thus, the 29 basins studied accounted for 41 native and 28 naturalized exotic species (S2 Table). Native species diversity ranged from 1 and 18 species per basin (Fig 2), while the naturalized species richness ranged between 0 to 16 species per basin (Fig 2).

Finally, we actualized the database according to the most recent taxonomical nomenclature. This considered the description of the *Olivaichthys viedmensis* (sin. *Diplomystes viedmensis*) found in the Patagonian region [55]; validation of *Aplochiton marinus* [56] and the species *Cheirodon pisciculus*, *C*. *galusidae*, *C*. *killiani*, and *C*. *australe* [57]; reassignment of *Basilichthys australis* to *B*. *microlepidotus* [58]; and the description of *Diplomystes incognitus* for central Chile [54].

### Historical and current assemblages

Using the fish species composition per basin (species × basins), we set up historical (pre-European) and current matrices (post-European), using ‘1’ and ‘0’ to denote presence and absence, respectively. The historical matrix considered the distribution of all native species as in pre-European times. Here, we assumed that the pre-European distribution of native fish was similar to that recorded at the beginning of the 20^th^ century. Although this assumption is usually invoked by most homogenization studies [59, 60], in Chile it is particularly well supported because–excepting an unique well-documented case (*Diplomystes chilensis*)–there are no records for extinction or translocation of native fishes [35–37, 40, 54, 61, 62]. Indeed, fishing upon native species has remained low up to the present time because they are not attractive for consumption, recreational fishing, ornamental or aquaculture purposes [34, 53, 54].

The current matrix considered current fish basin composition, including both native and exotic species. Given that the endemic *D*. *chilensis* is extinct [55], it was excluded from current matrix. Meanwhile, other native species just recently recorded as ‘absent’ or ‘possibly extirpated’ in certain tributaries [61–63], were not considered as extinct at basin scale.

### Evaluating homogenization

To determine if the Chilean basins have experienced a homogenization process in their freshwater fish fauna from historical to current times, we analyzed both the β-diversity and species turnover and nestedness using pairwise comparisons extracted from historical and current matrices.

We used the Sorensen dissimilarity index (β_sor_ = (*b* + *c*) · (2*a* + *b* + *c*)^-1^ [44]), Simpson index (β_sim_ = min{*b*,*c*} · (*a*+ min{*b*,*c*})^-1^ [44]), and β-nestedness index (β_nes_ = *a* · (max{*b*,*c*}–min{*b*,*c*}) · ((2*a* + min{*b*,*c*} + max{*b*,*c*}) · (*a* +min{*b*,*c*})^-1^ [44]) to calculate β-diversity, species turnover, and nestedness, respectively. For all these algorithms, *b* and *c* represent the richness recorded in any two basins compared (discarding the shared species), whereas *a* is the number of shared species between both. These indices range between 0 and 1, denoting minimum and maximum value for β-diversity (i.e., as a measure of the compositional changes among basins); species turnover (as a measure of the species replacement due to the existence of environmental and/or geographical barriers among basins); and species nestedness (as a measure of the differences in species richness when no species is replaced among basins). They were calculates using the historical and current distributional matrices, and we use them for calculate the differentials obtaining Δβ_sim_ (= βsim,Current− β_sim,Historical_), Δβ_nes_ (= βnes,Current− β_nes,Historical_), and Δβ_sor_ (= βsor,Current− β_sor,Historical_).

The current trend to homogenization was analyzed using two approaches. The first one provided a global overview of the basin set as a whole system. Here, we compared the β-indices for historical and current assemblages by means of a signed-ranks test (Wilcoxon matched-pairs test). This is a non-parametric test for two matched samples in two periods; we tested the observed differentials for Δβ_sim_, Δβ_nes_, and Δβ_sor_. Freshwater ichthyofauna transits to biotic homogenization when these differentials have negative bias (median < 0) [64, 65]. If differentials have positive bias (median > 0), then a biotic differentiation has occurred, which corresponds to a decrease in the compositional similarity between assemblages or, complementarily, an increase in β-diversity [64, 65]. Finally, when the differentials do not show any significant deviation from zero (median = 0) then no significant compositional changes has occurred [64, 65].

In a second analytical approach, we look if particular pairs of basins are homogenizing. Here, following the null model developed by Leprieur et al. [15], we reshuffled 10,000 times the distribution of the 28 exotic fish species present in the current assemblages; excepting the extinct *D*. *chilensis* [34], the distribution of the 40 extant native fish species was constant. This null model assumes that the distribution of exotic species changes randomly in the basins according to a fixed-equiprobable algorithm [66], which considers that the total number of basins in which each exotic species occurs is fixed, whereas the total number of exotic species per basin changes randomly (i.e., columns equiprobable) [15]. According to Leprieur et al. [15], this routine also considers the differences in colonization ability and/or human-induced propagule pressure among exotic species. Additionally, an equiprobable total of columns implies that all basins can be colonized by all the exotic species, and that the exotic fish species are distributed randomly among the 29 basins because all of them can colonize any basin [15]. Other biological and statistical implications about our null model have been discussed by Leprieur et al. [15].

We obtained 10,000 pseudovalues for β*_sim_, β*_nes_ and β*_sor_, and then calculated Δβ*_sim_ (= β*sim− β_sim,Historical_), Δβ*_nes_ (= β*nes− β_nes,Historical_), and Δβ*_sor_ (= β*sor− β_sor,Historical_). Thus, each basin pair showed a random distribution for Δβ*_sor_, Δβ*_sim_, and Δβ*_nes_ where the observed values (i.e., Δβ_sim_, Δβ_nes_, and Δβ_sor_) were compared. Biotic homogenization was defined as such when an observed Δβ value (calculated for a given basin pair) fell outside percentile < 2.5. Conversely, when the observed Δβ value was beyond the 97.5 percentile we interpreted it as biotic differentiation. In turn, observed Δβ between percentiles 2.5 and 97.5 were considered as un-changed [12].

### Predictors of β-diversity changes

To elucidate the putative drivers of fish homogenization in continental Chile we analyzed the role of area, altitudinal range, land use (agriculture, pasture, forest, mining, and urban uses), flow, elevation, and size of the human population (S1 Table). This information was obtained from Chile’s Ministerio del Medio Ambiente [53], with the exception of elevation (i.e., mean elevation in m), which was established as the elevation corresponding to the geometrical center drawn from the polygonal area for each basin. Geographical distance between basin pairs was calculated as the separation distance (km) between both geometrical centers considered by QGis (version 2.1.18). All of these variables were evaluated calculating the difference for a given basin pair, and then correlated with its corresponding change in Δβ_sim_ and Δβ_nes_. Finally, multivariate Mantel tests [67] were carried out to evaluate the statistical significance of these correlations.

### Future changes in β-diversity

We explored future homogenization trends based on probable composition of native and exotic species in the basins; here, we do not intend to predict the specific composition of freshwater fish for Chilean basins, but to grossly to establish the most likely trends for these assemblages under increased invasion and extinction rates.

Invasions were simulated considering the freshwater fish species with a demonstrated spread/colonization trait, as reported in the literature. We recognized the following categories: (a) freshwater fish species currently naturalized in Chile (S2 Table) that potentially can increase their distribution occupying other basins in the country [54], (b) introduction of “novel” exotic species currently not found in Chile but present in the Neotropical realm (S2 Table), and (c) introduction of “novel” exotic species currently absent in the Neotropical realm, but present in any other realm (Afrotropical, Nearctic, Australian, Oriental, and/or Palearctic; Table 1). These “novel” introductions of exotic fish species were extracted from the 43 more widely distributed species in the world, recorded by Toussaint et al. [68]. Three invasion levels were examined (Table 1): (a) spread of naturalized exotic species; (b) the same, but adding introduction of novel exotic species from the Neotropical realm, and finally, c) spread of naturalized exotic species, adding introduction of novel exotic species from any biogeographical realm. Thirty exotic species, currently not distributed in Chilean basins, could potentially be introduced and naturalized in the basins studied (S2 Table). Among them, 7 are present in the Neotropical realm and 23 in the other five biogeographical realms (S2 Table).

**Table 1:** Summary of future scenarios (n = 15) for 29 freshwater fish assemblages in Chile.

Future assemblages	Exotic species	Native species
Future 1	Spread of currently naturalized species (a)	Not applicable
Future 2	Spread of currently naturalized species + Introduction of exotic species from Neotropical realm (a + b)	Not applicable
Future 3	Spread of currently naturalized species + Introduction of exotic species from Neotropical realm + Introduction of exotic species from other realms (a + b + c)	Not applicable
Future 4	Not applicable	Extinction of ‘endangered’ native species (d)
Future 5	Not applicable	Extinction of ‘endangered’ + ‘vulnerable’ native species (d + e)
Future 6	Not applicable	Extinction of ‘endangered’ + ‘vulnerable’ native species + ‘near threatened’ native species (d + e + f)
Future 7	Spread of current naturalized species	Extinction of ‘endangered’ native species
Future 8	Spread of current naturalized species	Extinction of ‘endangered’ + ‘vulnerable’ native species
Future 9	Spread of current naturalized species	Extinction of ‘endangered’ + ‘vulnerable’ native species + ‘near threatened’ native species
Future 10	Spread of current naturalized species + Introduction of exotic species from Neotropical realm	Extinction of ‘endangered’ native species
Future 11	Spread of current naturalized species + Introduction of exotic species from Neotropical realm	Extinction of ‘endangered’ + ‘vulnerable’ native species
Future 12	Spread of current naturalized species + introduction of exotic species from Neotropical realm	Extinction of ‘endangered’ + ‘vulnerable’ native species + ‘near threatened’ native species
Future 13	Spread of current naturalized species + Introduction of exotic species from Neotropical realm + introduction of exotic species from other realms	Extinction of ‘endangered’ native species
Future 14	Spread of current naturalized species + Introduction of exotic species from Neotropical realm + introduction of exotic species from other realms	Extinction of ‘endangered’ + ‘vulnerable’ native species
Future 15	Spread of current naturalized species + Introduction of exotic species from Neotropical realm + introduction of exotic species from other realms	Extinction of ‘endangered’ + ‘vulnerable’ native species + ‘near threatened’ native species

These scenarios were generated by using exotic species introductions (three levels: a, b, c) and native species extinctions/extirpations (three levels: d, e, f).

The future geographic distribution for current and potential exotic species was obtained using MaxEnt [69]. This tool works with 19 bioclimatic variables, associated to monthly temperature and rainfall available from WorldClim [70]. For each non-native species, MaxEnt found basins where the environmental conditions are equivalent to current ranges [69], assigning potential presence where the focal species is currently absent [69]. Only 30 out of 43 species listed by Toussaint et al. [68] were predicted as potential colonizers for Chile (S2 Table), whereas 13 species did not show environmental matching with the Chilean basins. Although the native species were initially considered for these simulations, this was not possible because scarcity of collect records in basins, a necessary input for Maxent.

In addition, we simulated extinctions/extirpations of native species using their conservation categories [17], as established by Chile’s Ministerio del Medio Ambiente [53]. We considered official categories recognized by the Chilean authority for native freshwater fishes: (a) ‘endangered’ (EN; S2 Table), (b) ‘vulnerable’ (VU; S2 Table), and (c) ‘near threatened’ (NT; S2 Table). These categories were determined by limnologists and other specialists, who assigned this classification at basin or regional level (see S2 Table). This allowed us generating three levels of extinction/extirpation (Table 1): (a) all ‘endangered’ species were extinct or extirpated; (b) ‘endangered’ and ‘vulnerable’ were extinct or extirpated; and (c) ‘endangered’, ‘vulnerable’ and ‘near threatened’ were all extinct or extirpated. Since the translocation of native species has not been a practice in Chile (see Discussion) [53, 54], we do not include this factor in the generation of future assemblages. Because the current naturalization success, the exotic species were not considered as extinct in the future assemblages.

Thus, by using these different sets of native and exotic species we generated 15 future compositional scenarios for our 29 Chilean assemblages; these future assemblages were initially obtained simulating invasions, then extinctions/extirpations separately, and finally, analyzing their combined effects (Table 1). The future scenarios were contrasted with both historical and current assemblages to determine possible trends for β-diversity and its components. The homogenization/differentiation trends for simulated assemblages were analyzed using signed-ranks test (Wilcoxon matched-pairs test). To this purpose, values for β_sim,Future(1–15)_, β_nes,Future(1–15),_ β_sor,Future(1–15)_ were calculated, and then these were contrasted with equivalent values obtained for historical and current assemblages.

## Results

### Historical changes in β-diversity

**Fig 3:**
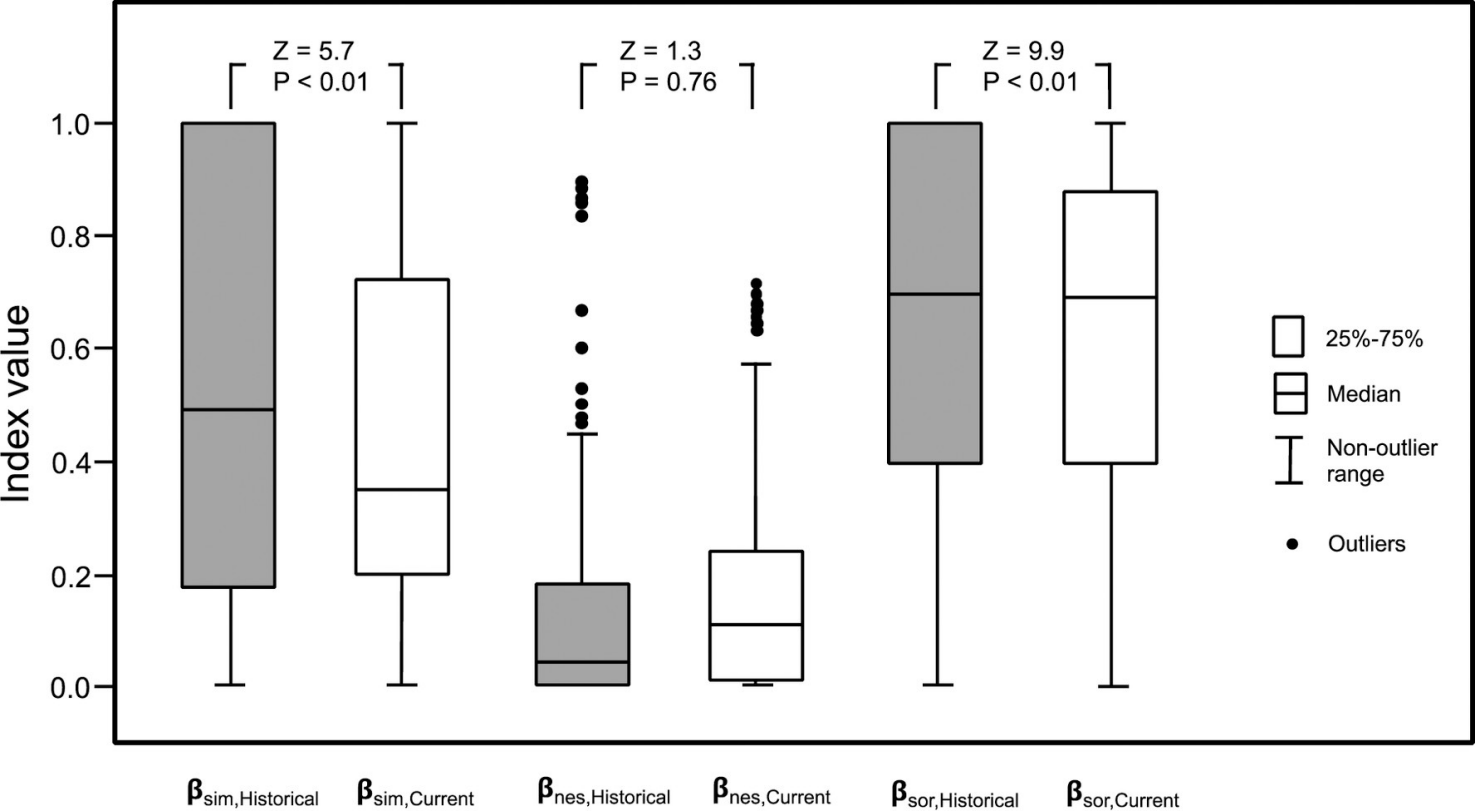
Box plot for distributions of turnover (β_sim,Historical_,β_sim,Current_), nestedness (β_nes,Historical_, β_nes,Current_) and β-diversity (β_sor,Historical_, β_sor,Current_) calculated for pairs among 29 basins. Distributions for these indices were compared considering historical and current freshwater fish assemblages.

Our analysis involving all of the basins as a whole system shows that they are in transit towards compositional homogenization of their freshwater ichthyofauna. Changes in turnover (Δβ_sim_ < 0) and β-diversity (Δβ_sor_ < 0) from historical to current time showed statistical differences when the basins were compared (Fig 3). Specifically, historical assemblages showed higher species turnover and β-diversity than current assemblages (Z = 5.7; Z = 9.9; both with P < 0.01; Fig 3). Nestedness (β_nes_) did not show significant differences between historical and current assemblages (Z = 0.3; P > 0.05; Fig 3).

When paired basins were compared, β_sim_ decreased (Δβ_sim_ < 0) in 156 out of 406 basin pairs (38%), with 76 significant cases (P < 0.025; S3 Table). Although these turnover changes involved several basins located throughout Chile, three northern basins (Camarones, Loa, and Huasco) accumulated the highest frequencies (see S3 Table). In turn, β_sim_ increased (Δβ_sim_ > 0) in 140 out of 406 basin pairs (34%) but none was statistically significant (S3 Table); Δβ_sim_ was null (Δβ_sim_ = 0) in 110 basin pairs (28%; S3 Table).

When β_nes_ was examined, 197 basin pairs (49%) showed a decrease (Δβ_nes_ < 0) from historical to current assemblages but none of these were statistically significant changes (S3 Table). A total of 94 comparisons (23%) showed no changes (Δβ_nes_ = 0; S3 Table), while 115 comparisons (28%) showed an increase (i.e., Δβ_nes_ > 0), where 63 basin pairs were significant (P > 0.975). These 63 cases were located in three northern basins (Chungará, Camarones, and Loa, respectively; S3 Table).

Finally, Δβ_sor_ decreased (Δβ_sor_ < 0) in 197 basin pairs (48%), where 111 were significant changes (P < 0.025); these comparisons were widely distributed throughout Chile, but Loa, Copiapó, Huasco, Elqui, Limarí, and Choapa accumulated the highest frequencies (S3 Table). Δβ_sor_ was equal to zero in 95 comparisons (24%), and was greater than zero (Δβ_sor_ > 0) in 114 comparisons (28%).

### Species distributions and occupancy

**Fig 4:**
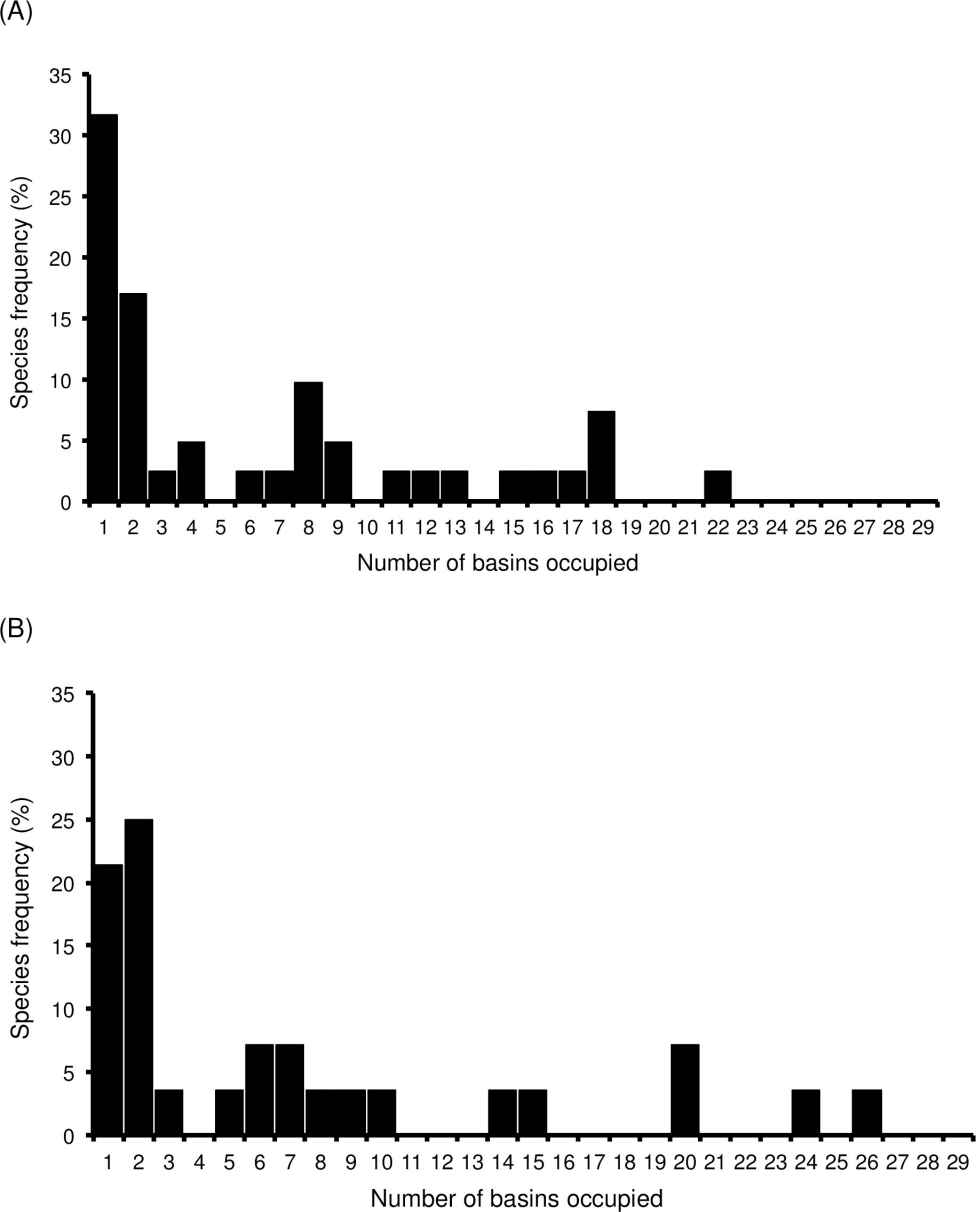
Basin occupancy (%) for 41 native and 28 naturalized freshwater fish species distributed in 29 basins in continental Chile.

Because changes in β-diversity result from species distributions, we analyzed the fish occupancy, founding about 32% of the native species were distributed only in one basin (Fig 4A). Native *Galaxias maculatus* was the most widely distributed fish, which was present in 22 basins, followed for *Basilichthys microlepidotus*, *Geotria australis* and *Mordacia lapicida*, the three of them present in 18 basins (S2 Table). Among exotic species, ca. 21% were distributed in only one basin (Fig 4B), while *Oncorhynchus mykiss*, *Salmo trutta*, *Gambusia affinis*, *G*. *holbrooki*, and *Cyprinus carpio* were the most widely distributed, being present in 26, 24, 20, 20 and 15 basins, respectively (S2 Table). Native and naturalized species occupancies did not show statistical differences (Kolmogorov-Smirnov test, Z = 1.5; P > 0.75).

### Predictors for β-diversity changes

Among the factors that were correlated with Δβ_sim_, Δβ_nes_, and Δβ_sor_ only changes in area, elevation, and geographical distance between basins, exhibited significant effects (Table 2). For Δβ_sim_ and Δβ_sor_, all these correlations were negative (r < 0; Table 2), while being positive for Δβ_nes_ (r > 0; Table 2). The diverse land uses (i.e., agriculture, pasture, forest, urban, mining), the human population size, and flow did not show significant correlations with Δβ_sim_, Δβ_nes_ neither Δβ_sor_ (Table 2).

**Table 2:** Correlations between differentials of turnover (Δβ_sim,Current_ = β_sim,Current_ –β_sim,Historical_), nestedness (Δβ_nes,Current_ = β_nes,Current_− β_nes,Historical_), and β-diversity (Δβ_sor,Current_ = β_sor,Current_− β_sor,Historical_) with environmental differentials for factors considered.

Factors	Δβ_sim_	Δβ_nes_	Δβ_sor_
Area	-0.384*	0.312*	-0.311*
Elevation	-0.543*	0.506*	-0.385*
Geographical distance	-0.363*	0.344*	-0.266*
Agriculture	0.055	0.002	0.177
Pasture	-0.023	-0.003	-0.044
Forest	0.082	-0.031	0.122
Urban	0.023	0.034	0.120
Mining	0.099	-0.031	0.132
Human population size	0.012	0.044	0.098
Flow	0.093	-0.025	0.113

(*) Indicates statistical significance (P < 0.975 or P > 0.025; Multivariated Mantel-test).

### Future changes in β-diversity

**Fig 5:**
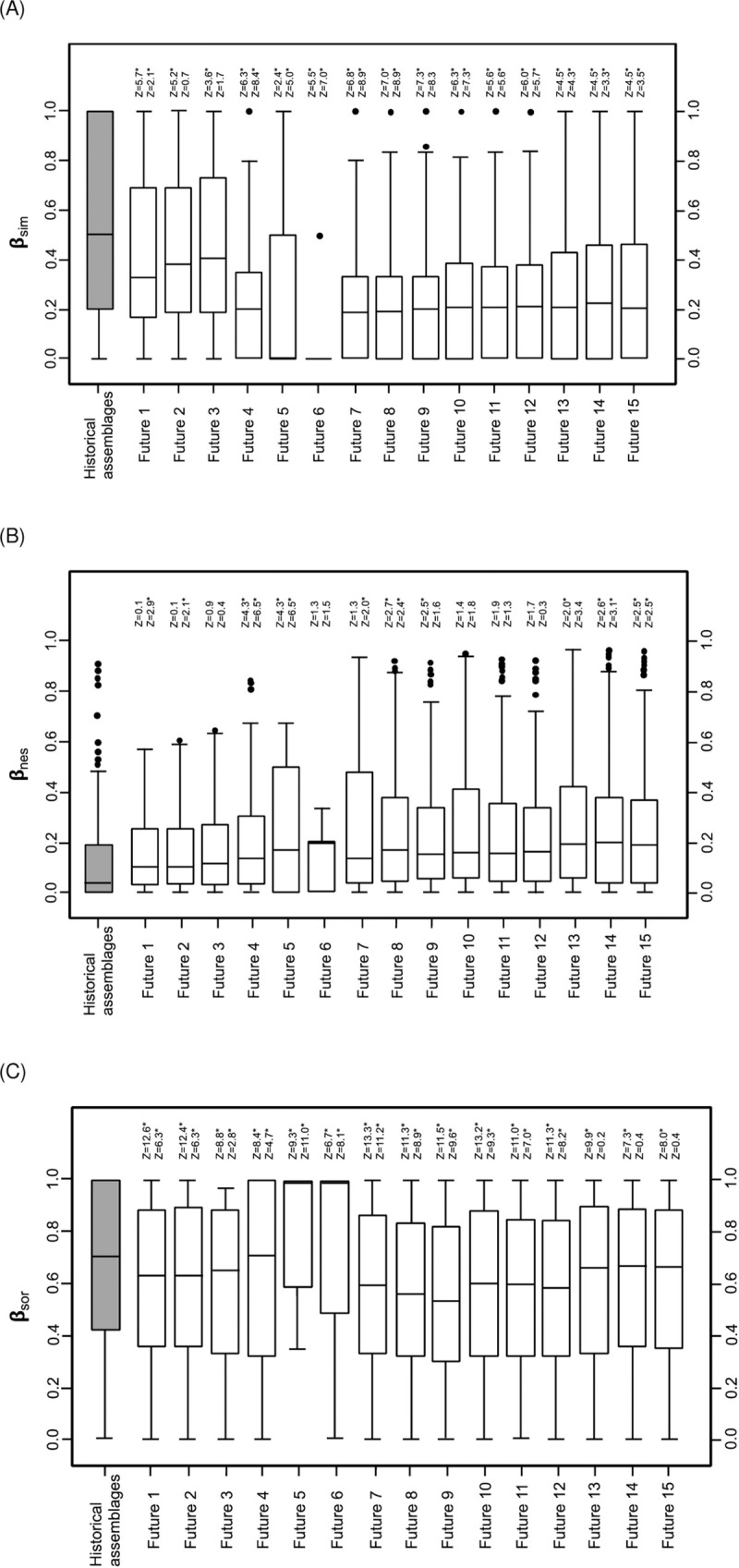
Box plot for distributions of (A) turnover (β_sim,Future(1–15)_), (B) nestedness (β_nes,Future(1–15)_) and (C) β-diversity (β_sor,Future(1–15)_) calculated for pairs among 29 basins. The distributions were compared by signed-ranks tests, contrasting each assemblage with historical and current assemblages. The values of each statistic (Z) and significance (*: P < 0.05) are shown for these comparisons, respectively. The distributions of β_sim,Historical_, β_nes,Historical_, β_sor,Historical_ (gray boxes) were included as reference.

The 15 future assemblages showed a significant decrease for β_sim_ and β_sor_ from historical and current values (Fig 5A and 5C), and in turn, a significant increase of β_nes_ occurred in 7 out 15 assemblages (Fig 5B). Additionally, when the future values for β-diversity (β_sim,Future(1–15)_), nestedness (β_nes,Future(1–15)_), and turnover (β_sor,Future(1–15)_) were respectively subtracted from current values (β_sim,Current_, β_nes,Current_, β_sor,Current_) obtaining the differentials (Δβ_sim,Future(1–15)_, Δβ_nes,Future(1–15)_, and Δβ_sor,Future(1–15)_), these were higher than those calculated between current and historical times (Δβ_sim_: F = 143.9; β_nes_: F = 98.6; β_sor_: F = 102.3; all P < 0.001; ANOVA).

According to our simulations, introduction of novel exotic species and extinctions/extirpations of native ones were statistically significant for modifying the β-indices; however, the effects of extinctions/extirpations upon the three β-indices were significantly higher when the differential distributions were compared (β_sim_: F = 180.9; β_nes_: F = 199.8; β_sor_: F = 261.5; all P < 0.001; ANOVA). These results indicate that biotic homogenization is the most likely future trend for the studied basins, because β_sim_ and β_sor_ diversity would tend to decrease significantly, whereas β_nes_ do so in particular scenarios (Fig 5B); additionally, these changes will be promoted by the intensification of extinctions, and to a lesser extent, by the increase in invasions.

## Discussion

Our analysis shows that currently the Chilean basins are in transit towards compositional homogenization of their freshwater ichthyofauna. This trend results mainly because fish species deliberately introduced are currently naturalized in the basins, concomitantly with a very low extinction rate and a null translocation of native species (S2 Table). Thus, these basins have decreased significantly their distributional β-diversity (β_sor_) from 0.684 to 0.623 on the average, implying a significant decrease equal to 8.9% (100 ·Δβ_sor,Current_ · (β_sor,Historical_)^-1^ = 100 · (-0.061) · (0.684)^-1^). In addition, species turnover (β_sim_) significantly decreased from 0.546 to 0.462 (15.4%), while nestedness (β_nes_) showed no significant change.

Both turnover as nestedness have been recognized as additive components of β-diversity [44]. They are quantified by using metrics related to different spatial processes [44, 47, 71, 72]. In the present study, species turnover denotes a reduction of the ‘true’ spatial turnover (*sensu* Baeten et al. [49]), without effect of the differences in species richness among basins [49, 71]. Thus, a significant decrease of turnover implies the dilution of the underlying biogeographic barriers, a process indicative of biotic homogenization [47, 71]; interestingly, this pattern is due to only few exotic species (see below) because most part of them are distributed as native ones (Fig 4). In turn, the nestedness component accounts for the differences in composition or species richness when no species is replaced among basins [47, 71]. In the Chilean case, the nestedness distribution does not show significant changes when historical and current times were compared; this is mainly because most of the native species (98%) have maintained their geographical distribution and most of the exotic species (approx. 70%) are distributed as native ones (Fig 4).

These findings expand on previous studies reporting incipient homogenization among the Chilean ichthyological provinces [41, 42] and within the Neotropical realm [15, 73]. A first implication of our results is that the Chilean basins share the generalized (global) homogenization trends, also recorded in other countries such as Australia [74], China [18–20], Japan [17], Portugal-Spain [13, 75], European countries [76], and United States [11, 12]. The importance of the analyses based on countries as study units lies in that–if biotic homogenization is an undesired byproduct of global biotic change [10]–it would be more feasible to stop or to revert, because countries can take appropriate actions and use political tools for their territorial management [77].

According to previous studies, Neotropical basins are in transit toward biotic homogenization [15, 73]; particularly so those located in central Chile (30–37^o^ S; see Fig 1), which homogenizing together with other Mediterranean basins around the world [40]. However, the specific mechanisms involved in the Chilean basins show different susceptibility, magnitude and rate of biotic homogenization. First, native species richness of the Chilean basins (9 species per basin, on average) is lower than other Neotropical basins (25 species per basin), permitting that in Chile a small number of exotic species may modify the β-diversity among basins [73]. Second, the average number of exotic species per basin inside the Neotropical realm (2.0 exotic species per basin) [15, 73] is lesser than that recorded for the Chilean basins (6.8 exotic species per basin; Table 2). It is important to highlight that the number of naturalized species in Chile is high regarding its native richness (approximately 70%), a reason why two biogeographic areas (i.e., central Chile and southern South America) have been considered as invasion hotspots at global level [15, 40, 73]. Third, Neotropical basins contain a high proportion (61%) of translocated species [73]. In Chile, species translocation is not a practice that has been previously implemented [see also 39, 54, 78, 79]; only the recent construction of irrigation canals in a small number of basins may promote the future exchange of species between them [80]. The reduced translocation rate of native fishes is related to their not being attractive for human consumption, recreational fishing, ornamental or aquaculture purposes [54, 61]. Fourth, a commonality: the Neotropical realm and Chilean basins show a similar extinction/extirpation rates, ranging between 0 and 1 species per basin [15, 73]. Thus, although Neotropical basins transit toward taxonomic homogenization, the mechanisms implicated in the Chilean basins suggest a higher rate of homogenization process.

Currently, Chilean basins harbor 28 exotic species that came from diverse biogeographic origins. These species have been introduced intentionally, mainly from aquaculture, recreational fisheries, and as ornamental pet species [54, 78, 79]; currently, they are naturalized in several [39, 54, 78, 79]. The most widely distributed species are represented by *O*. *mykiss*, *S*. *trutta*, *G*. *affinis*, *G*. *holbrooki*, and *C*. *carpio*, whose occupancies range from 15 to 26 basins (S2 Table). Twenty-seven out of 29 basins studied here had at least one exotic species (S2 Table), which follows a similar latitudinal richness pattern as native ones, being low in northern Chile (1–5 species per basin), but increasing from 28°S onwards (6–16 species per basin). On the other hand, native species show a similar distribution pattern, with species richness increasing from northern (1–5 species per basin) towards southern basins, where it reaches between 13–18 species per basin, and then decrease to 5–10 species per basin. Interestingly, twenty three out of 28 exotic species came from Palearctic and/or Nearctic realms, excepting *Australoheros facetus*, *Cheirodon interruptus*, *Cnesterodon decemmaculatus*, *Jenynsia multidentata*, and *Odontesthes bonariensis*. In addition, most of these exotic species belong to genera and families previously not represented in Chilean basins, reaching 40.1% of the current species richness in Chile.

As a homogenizing mechanism, extinctions have had reduced importance from historical to current assemblages; the only recognized extinction in a Chilean basin is *D*. *chilensis* [55]. Other species have been reported as local extinctions, a reason why they were not considered in the present study (their extirpation from rivers or tributaries does not imply extinction around the entire basin).

Complementarily, our analysis based on paired comparisons indicates that significant decrease for β-diversity and turnover were concentrated in a reduced number of basin pairs, involving between 38 and 56% of the paired comparisons, respectively. Although the homogenized basins widely distributed, the more frequent homogenized were located in north-central Chile (i.e., S3 Table). Here, β-diversity and turnover decreased significantly as consequence of poor native richness (1–6 species per basin), and the presence of four widely distributed exotic species in Chile (*G*. *affinis*, *G*. *holbrooki*, *O*. *mykiss*, and *S*. *trutta*). Other studies based on paired comparison also showed that a small number of basins undergoing homogenization [5, 10]. In addition, the homogenization of Chilean freshwater fish fauna is geographically structured because north-central basins accumulated a greater number of significant changes in β-diversity, than did southern ones.

The taxonomic homogenization reported here requires to be complemented with other facets of biodiversity in Chilean basins. For example, fish samplings show that abundance of exotic species is higher than that of native ones [80, 81]. These findings indicate that taxonomic homogenization can be even more intense when metrics based on abundance or equitability are used. These abundance patterns can have important ecological consequences for the structure of the 29 basins studied. Thus, a significant advance in our comprehension of biotic homogenization requires studies including population abundances and not only taxonomic species lists [10]. In this line, current as well as future scenarios need to be evaluated in regard to how other facets of biodiversity can be modified as a result of invasion/extinction imbalance [10, 60, 82]. Both native as exotic species present in Chile show differential morphofunctional traits (i.e., body sizes, trophic habits, reproduction, behavior, etc.), thus affecting the functional structure of the fish assemblages within and among basins [82]. As was previously noted, the most part of exotic species belong to genera, families, and orders not represented in the native ichthyofauna. Therefore, functional and phylogenetic diversity as different dimensions of the biotic change demand further studies.

Current changes in turnover and nestedness were correlated with geographical parameters such as area, elevation, and geographical distance. In all cases, correlations for turnover were negative while being positive for nestedness, evidencing the expected effect of spatial distances upon components of β-diversity [46]. The absence of significant correlations between components of β-diversity and land-use variables (i.e., agriculture, pasture, forest, mining, and urban uses) [83] seems noteworthy. However, these findings can be explained basically because the introduction of exotic species has been fueled mainly by aquaculture, recreational fisheries, and ornamental pet species, without attempts for basin management [84]. Recently, Habit et al. [83], analyzing patterns of native and exotic richness in Chilean Patagonian lakes, reported absence of correlation between human activities and fish species richness, although geographical connectivity appears to determine spread of exotic fish species among these Chilean lakes [83].

Similarly to other studies considering future scenarios [17, 22, 31], our compositional prospects suggest that biotic homogenization for Chilean basins should increase in the future, although at unknown rate. Nevertheless, our simulated scenarios must be considered carefully because they depend on specific assumptions. In Chile, the introduction of new exotic species is currently regulated by two institutions (the Under-Secretariat for Fisheries, SUBPESCA, in Spanish; and the National Fisheries Service, SERNAPESCA, in Spanish) [39], whose control mechanisms reduce the probability that new exotic species will be accepted. Thus, the introduction of 30 novel species is unlikely, at least in the short term. On the other hand, recent studies have emphasized the need to adopt effective procedures for the conservation of native species [38, 61, 79, 85], and although it is unlikely that extinction rate will reach the simulated magnitudes–at least in the short term–this factor constitutes the most imminent threat to freshwater fish diversity in Chile. Particularly worrying are Ascotán and Isluga basins (in the Andean plateau), because their native species (*Orestias ascotanensis* and *O*. *cf*. *agastii* and *Thrichomycterus rivulatus*, respectively) are considered as endangered (S2 Table). Because of environmental conditions (highly saline, slow flow) [84], remoteness [53], and restricted accessibility [53], it is unlikely that exotic fish species will be naturalized there. Therefore, these two Chilean basins could completely lose their freshwater ichthyofauna.

In the future scenarios for Chilean basins, the relative importance of invasions as homogenizing mechanisms should decrease, and conversely, the role of extinctions/extirpations should increase. Indeed, the effects of native extinction/extirpation upon future β-diversity, future turnover and future nestedness were significantly greater than spread of naturalized and/or introduction novel exotic species. These results differ from those obtained by Villéger et al. [86], who found greater importance of invasions than extinctions as determining homogenization at regional and global levels including the Neotropical realm. This discrepancy is ascribable to geographical distribution of the current species richness (i.e., fish species), and basin susceptibilities to species extinctions and introductions. In Chile, for example, the high endemism (80%) is associated with a high geographical turnover [53], so future extinctions (involving the most of part of endemic species) will contribute to the loss of biodistinctiveness among basins, increasing homogenization [12]. We highlights these findings as relevant for the conservation of β-diversity in the Chilean basins [60].

In summary, our results indicate that the major basins of continental Chile are in a process of biotic homogenization in ichthyofauna, trend shared with other basins of the Neotropical region and the World. Nevertheless, the mechanisms promoting it in Chile differ in magnitude from those reported in other regions. According to simulated trends, biotic homogenization should increase, especially in response to extinction of native species that are currently of conservation concern.

## Supporting information

S1 File References used to analyze the geographical distribution of freshwater fishes in Chilean basins. (DOCX)

S1 Table Twenty-nine Chilean basins and their geographical characteristics: Total area (km^2^), elevation (m), land use (area used for agriculture, pasture, forest, urban, and mining), human population size (current inhabitants in basins) and water flow (m^3^·s^-1^). (XLSX)

S2 Table Data matrix with freshwater fish species, origin, status and occurrence at studied basins. The information is shown in brackets according to presence/absence (1 and 0, respectively) in historical, current, and future assemblages. For native and naturalized species, brackets were ordered as: [historical presence, current presence, presence in ‘Future 1’ assemblage], presence in assemblage ‘Future 2’, presence in ‘Future 3’ assemblage]. For exotic species potentially successful colonizers to the Chilean basins, the information was ordered as: [historical, current, presence in ‘Future 4’ assemblage, presence in ‘Future 5’ assemblage, presence in ‘Future 6’ assemblage]. By using this information other nine assemblages were conformed.
(XLSX)

S3 Table Matrices for Δβ_sim_ (Matrix A: β_sim,Current_− β_sim,Historical_), Δβ_nes_ (Matrix B: β_nes,Current_− β_nes,Historical_) and Δβ_sor_ (Matrix C: β_sor,Current_− β_sor,Historica_) calculated on basin pairs of 29 Chilean watersheds. Basins were latitudinally ordered (see Fig 1). Chungará (Chu), Lauca (Lau), Camarones (Cam), Isluga (Isl), Ascotán (Asc), Loa (Loa), Copiapó (Cop), Huasco (Hua), Elqui (Elq), Limarí (Lim), Choapa (Cho), Aconcagua (Aco), Maipo (Mai), Rapel (Rap), Mataquito (Mat), Maule (Mau), Itata (Ita), Andalién (And), Biobío (Bio), Imperial (Imp), Toltén (Tol), Valdivia (Val), Bueno (Bue), Maullín (Mal), Palena (Pal), Cisnes (Cis), Aysén (Ays), Baker (Bak), and Serrano (Ser). Bold italic numbers indicate significant statistical differences (P < 0.025 or P > 0.975).
(XLSX)
