# Supplementary material for: Partitioning β-diversity reveals that invasions and extinctions promote the biotic homogenization of Chilean freshwater fish fauna
Source: PLoS One. 2020 Sep 8;15(9):e0238767. doi: 10.1371/journal.pone.0238767 (PMC7478641; doi:10.1371/journal.pone.0238767)
Supplement: S1 File — (DOCX) [file pone.0238767.s001.docx]

S1. References of the distribution of native and exotic species in the Chilean basins.

1. Acuña, P., Vila, I., Pardo, R., Comte, S., 2005. Caracterización espacio-temporal del nicho trófico de la fauna íctica andina del río Maule, Chile. Gayana 69, 175-179. DOI: 10.4067/S0717-65382005000100022.
2. Aedo, J., Belk, M., Habit, E., 2009. Geographic variation in age, growth and size structure of *Percilia irwini* from south-central Chile. Journal of Fish Biology 74, 278–284. DOI: 10.1111/j.1095-8649.2008.02113.x.
3. Alò, D., Correa, C., Arias, C., Cárdenas, L., 2013. Diversity of Aplochiton Fishes (Galaxiidea) and the Taxonomic Resurrection of *A*. *marinus*. Plos one 8(8), e71577. DOI: 10.1371/journal.pone.0071577.
4. Arismendi, I., Penaluna, B., 2009. Peces nativos en aguas continentales del Sur de Chile. Imprenta América Ltda.
5. Arismendi, I., Soto, D., Penaluna, B., Jara, C., Leal, C., León-Muñoz, J., 2009. Aquaculture, non-native salmonid invasions, and associated declines of native fishes in lakes of the northern Chilean Patagonia. Freshwater Biology 54,1135–1147. DOI: 10.1111/j.1365-2427.2008.02157.x.
6. Arratia, G., 1981. Géneros de peces de aguas continentales de Chile. Publicación Ocasional del Museo Nacional de Historia Natural 34, 3-108.
7. Arratia, G. 1982. A review of freshwater percoids from South America (Pisces, Osteichthyes, Perciformes, Percichthyidae, and Percillidae). Senckenberg Naturforsch Gesellsch 540, 1-52.
8. Arratia, G., 1983. *Trichomycterus chungaraensis* n. sp. and *Trichomycterus laucaensis* n. sp. (Pisces, Siluriformes, Trichomycteridae) from the high Andean range. Studies on Neotropical Fauna and Environment 18, 65-87.
9. Arratia, G., 1987. Description of the primitive family Diplomystidae (Siluriformes, Teleostei, Pisces): morphology, taxonomy and phylogenetic implications. Bonner zoologische Monographien 24, 5-120.
10. Arratia, G., 1992. Development and variation of the suspensorium of primitive catfishes (Teleostei: Ostariophysi) and their phylogenetic relationships. Bonner zoologische Monographien 32, 1-149.
11. Arratia, G., Chang, A., Menu-Marque, S., Rojas, G., 1978. About *Bullockia* gen. nov,.*Trichomycterus mendozensis nov*.*sp*. and revision of the family Trichomycteridae (Pisces, Siluriformes). Studies on Neotropical Fauna and Environment 13, 157-194.
12. Arratia, G., Menu-Marque, S., 1981. Revision of the freshwater catfishes of the genus *Hatcheria* (Siluriformes, Trichomycteridae) with commentaries on ecology and biogeography. Zoologische Anzeiger 207(1-2), 88-111.

1. Arratia, G., Quezada-Romegialli, C., 2017. Understanding morphological variability in a taxonomic context in Chilean diplomystids (Teleostei: Siluriformes), including the description of a new species. PeerJ 5, e2991. DOI: 10.7717/peerj.2991.
2. Arratia, G., Vila, I., Lam, N., Guerrero, C.J., Quezada-Romegialli, C., 2017. Morphological and taxonomic descriptions of a new genus and species of killifishes (Teleostei: Cyprinodontiformes) from the high Andes of northern Chile. Plos one 12(8), e0181989. DOI: 10.1371/journal.pone.0181989.
3. Astorga, M., Valenzuela, C., Arismendi, I., Iriarte, J., 2008. Salmones Chinook asilvestrados en el norte de la Patagonia chilena: ¿se originan desde escapes de cultivo?. Biología Marina y Oceanografía 43, 669-674. DOI: 10.4067/S0718-19572008000300025.
4. Basulto, S., 2003. El largo viaje de los salmones: una crónica olvidada, propagación y cultivo de especies acuáticas en Chile. Editorial Maval, Santiago de Chile. 299 pp.
5. Beltrán, M., Muñoz, C., Ibarra, J., Habit, E., 2012. Análisis de la dieta de *Diplomystes* (Siluriformes: Diplomystidae) de Chile Gayana 76, 102-111. DOI: 10.4067/S0717-65382012000300003.
6. Bravo, S., 2012. Guía de pesca recreativa para el Río Palena. Gobierno Regional de los Lagos. Chile.
7. Campos, H., 1970. Introducción de especies exóticas y su relación con los peces de agua dulce de Chile. Noticiario Mensual del Museo Nacional de Historia Natural, Chile 14 (162), 6-9.
8. Campos, H., 1979. Avance en el estudio sistemático de la familia Galaxiidae (Osteichthyes: Salmoniformes). Archivos de Biología y Medicina Experimentales 12, 107-118.
9. Campos, H., 1982. Sistemática del género *Cheirodon* (Pisces: Characidae) en Chile con descripción de una nueva especie. Análisis de multivarianza. Studies on Neotropical Fauna and Environment 17, 129-162.
10. Campos, H., 1985. Distribution of the fishes in the Andean rivers in the South of Chile. Archives Hydrobiology 104 (2), 169 - 191.
11. Campos, H., Gavilán, J.F., Murillo, V., Alarcón, P., 1996. Presencia de *Cheiredon australe* (pisces: characiodae) en lago Tarahuin (Isla Grande de Chiloe, 42° s, Chile) y su significado zoogeografico. Medio Aambiente 13 (1), 69-79.
12. Campos, H., Dazarola, G., Dyer, B., Fuentes, L., Gavilán, L., Huaquín, G., Martínez, R., Meléndez, G., Pequeño, F., Ponce, V., Ruiz, W., Sielfeld, W., Soto, D., Vega, R., Vila, I., 1998. Categorías de conservación de peces nativos de aguas continentales de Chile. Boletín del Museo Nacional de Historia Natural 47, 101-122.
13. Colin, N., Piedra, P., Habit, E., 2012. Variaciones espaciales y temporales de las comunidades ribereñas de peces en un sistema fluvial no intervenido: Río San Pedro, Cuenca del Río Valdivia (Chile). Gayana, 76, 36-44. DOI: 10.4067/S0717-65382012000100003.
14. Cuevas, C.C., Campos, H., Busse, K., 1999. Cytotaxonomic Studies on Chilean Galaxiid Fishes. The Karyotypes, C-bands, Ag-NORs and Hybrids of *Brachygalaxias gothei* and *B*. *bullocki* (Osteichthyes: Galaxiidae). Cytologia, 64(4), 379-385. DOI: 10.1508/cytologia. 64.379.
15. Cussac, V., Ortubay, S., Iglesia, G., Milano, D., Lattuca, M.E., Barriga, J.P., Battini, M., Gross, M., 2004. The distribution of South American galaxiid fishes. The role of biological traits and post- glacial history of Chile. Boletín del Museo Nacional de Historia Natural 48, 61–75. DOI: 10.1046/j.0305-0270.2003.01000.x.
16. Dazarola, G., 1972. Contribution a l' ètude de la faune ichtyologique de la region Valparaíso Aconcagua (Chili). Annals de Limnologie, Paris 8 (1), 87-100.
17. De Buen, F., 1959. Los peces exóticos en las aguas dulces de Chile. Investigación Zoológicas Chilena 5: 103-137.
18. De la hoz, E., 1994. Aspectos cinemáticos del mecanismo de mordida premaxilar en los géneros *Cauque*, *Basilichthys* y *Austrimenidia*. Investigaciones marinas 22, 31-37. DOI: 10.4067/S0717-71781994002200003.
19. DSS Ambiente, 2009. Análisis de impacto económico y social de anteproyecto de normas secundarias de calidad – cuenca río Mataquito. Ministerio de Obras Públicas Dirección General de Aguas.
20. Duarte, W., Feito, R., Jara, C., Moreno, C., Orellana, A.E., 1971. Ictiofauna del sistema hidrográfico del río Maipo. Boletín del Museo Nacional de Historia Natural, Chile 32, 227-268.
21. Dyer, B., 1997. Phylogenetic revision of Atherinopsinae (Teleostei, Atherinopsidae), with comments on the systematics of the South American freshwater genus *Basilichthys* Girard. Miscellaneous Publications, Museum of Zoology, University of Michigan 187, 1-64.
22. Dyer, B., 2000. Revisión Sistemática de los Pejerreyes de Chile (Teleostei,Atheriniforme sistematyc review of the fishes of Chile (teleostei, atheriniformes). Estudios oceanológicos 19, 99-127.
23. Eigenmann, C., 1927. The fresh-water fishes of Chile. Memoirs of the National Academy of Sciences 22, 1-63.
24. Figueroa, R., Suarez, M., Andreu, A., Ruiz, V., María, R., Abarca, V., 2009. Caracterización ecológica de humedales de la zona semiárida en Chile central. Gayana 73, 76-94. DOI: 10.4067/S0717-65382009000100011.
25. Fowler, H., 1951. Analysis of the fishes of Chile. Revista Chilena de Historia Natural 63, 263-326.
26. Fuentes, L., Vila, I., Contreras, M., 2005. Temperature, energy acquisition and energy use in the Chilean silversidae *Basilichthys australis* Eigenmann. Revista chilena de historia natural 78, 303-311. DOI: 10.4067/S0716-078X2005000200014.
27. Gallardo, J., 2006. Tesis de grado de Biólogo Marino; “Caracterización de la colonización de *Oncorhynchus tshawytscha*(Walbaum; 1792) en el Río Cobarde, XI Región de Aysén”. UACH. Valdivia.
28. García, A., Jorde, K., Habit, E., Caaman, D., Parra, O., 2011. Downstream environmental effects of dam operations: changes in habitat quality for native fish species. River Research and Applications 27, 312–327. DOI: 10.1002/rra.1358.
29. García, A., González, J., Habit, E., 2012. Caracterización del hábitat de peces nativos en el río San Pedro (cuenca del rio Valdivia, Chile). Gayana 76, 24-35. DOI: 10.4067/S0717-65382012000100004.
30. Habit, E., Victoriano, P., Campos, H., 2003. Ecología trófica y aspectos reproductivos de *Trichomycterus areolatus* (Pisces, Trichomycteridae) en ambientes lóticos artificiales. Revista de Biología Tropical 53, 195-210.
31. Habit, E., Victoriano, P., Rodríguez-Ruiz, A., 2003. Variaciones espacio-temporales del ensamble de peces de un sistema fluvial de bajo orden del centro-sur de Chile. Revista Chilena de Historia Natural 76(1), 3-14. DOI: 10.4067/S0716-078X2003000100001.
32. Habit, E., 2005. Aspectos de la biología y hábitat de un pez endémico de Chile en peligro de extinción (*Diplomystes nahuelbutaensis* Arratia 1987. Scielo Interciencia 30, 1-10.
33. Habit, E., Dyer, B., Vila, I., 2006. Estado de conocimiento de los peces dulceacuícolas de Chile. Gayana, 70, 100–113. DOI: 10.4067/S0717-65382006000100016.
34. Habit, E., Victoriano, P., 2006. Peces de agua dulce de la Cordillera de la Costa. Gayana Especial, 10-23.
35. Habit, E., Belk, M., 2007. Threatened fishes of the world: *Percilia irwini* (Eigenmann 1927) (Perciliidae). Environmental Biology of Fishes 78(3), 213-214. DOI: 10.1007/s10641-006-0014-4.
36. Habit, E., Belk, M., Parra, O., 2007. Response of the riverine fish community to the construction and operation of a diversion hydropower plant in central Chile. Aquatic conservation: Marine and Freshwater Ecosystems 17(1), 37-49. DOI: 10.1002/aqc.774.
37. Habit, E., Belk, M., Victoriano, P., Jaque, E., 2007. Spatio-temporal distribution patterns and conservation of fish assemblages in a Chilean coastal river. Biodiversity and Conservation 16(11), 3179-3191. DOI: 10.1007/s10531-007-9171-9.
38. Habit, E., Piedra, P., Ruzzante, D., Walde, S., Belk, M., Cussac, V., Gonzalez, J., Colin, N., 2010. Changes in the distribution of native fishes in response to introduced species and other anthropogenic effects. Global Ecology and Biogeography,19, 697–710. DOI: 10.1111/j.1466823.2010.00441.X
39. Habit, E., Gonzalez, J., Ruzzante, D., Walde, S., 2012. Native and introduced fish species richness in Chilean Patagonian lakes: inferences on invasion mechanisms using salmonid-free lakes. Diversity and Distributions 1–13.
40. Habit, E., Victoriano, P., 2012. Composición, origen y valor de conservación de la Ictiofauna del Río San Pedro (Cuenca del Río Valdivia, Chile). Gayana Especial, 10-23. DOI: 10.4067/S0717-65382012000100002.
41. Habit, E., González, J., Ortiz-Sandoval, J., Elgueta, A., Sobenes, C., 2015. Efectos de la invasión de salmónidos en ríos y lagos de Chile. Revista Ecosistemas, 24(1), 43-51. 19, 697–710. DOI:10.1111/j.1466823.2010.00441.X.
42. Ibarra, J., Habit, E., Barra, B., Solís, K., 2011. Juveniles de salmón Chinook (*Oncorhynchus tshawytscha* Walbaum, 1792) en ríos y lagos de la PatagoniaChilena. Gayana 75, 17–25. DOI: 10.4067/S0717-65382011000100002.
43. Iriarte, J., Lobos, G., Jaksic, F., 2005. Invasive vertebrate species in Chile and their control and monitoring by governmental agencies. Revista Chilena de Historia Natural 78, 143–154. DOI: 10.4067/S0716-078X2005000100010.
44. Marr, S.M., Olden, J.D., Leprieur, F., Arismendi, I., Ćaleta, M., Morgan, D.L., Nocita, A., Šanda, R., Tarkan, A.S., García-Berthou, E., 2013. A global assessment of freshwater fish introductions in mediterranean-climate regions. Hydrobiologia, 719, 317-329. DOI:10.1007/s10750-013-1486-9.
45. Medel, O., Pérez, E., Saavedra, E., 2010. Fortalecimiento de la institucionalidad reguladora y fiscalizadora del sector acuícola en Chile URL: http://www.sociedadpoliticaspublicas.cl/archivos/BLOQUE2/Politicas_Publicas_y_Sectores_Productivos/Fortalecimiento_de_la_Institucionalidad_Reguladora_y_Fiscalizadora_del_Sector_Acuicola_en_Chile.pdf/ Accesado: Marzo 25, 2017.
46. Ministerio de Agricultura Corporación Nacional Foresta. Plan de manejo reserva nacional laguna Torca. República de Chile.
47. Montory, M., Habit, E., Bahamonde, P., Fernandez, P., Grimalt, J., Saez, K., Rudolph, I., BarraM R., 2011. Polychlorinated biphenyls in farmed and wild *Onchorhynchus kisutch* and *Onchorhynchus mykiss* from the Chilean Patagonia. Environ Sci Pollut Res Int. 18:629–637. DOI: 10.1007/s11356-010-0408-x.
48. Morey, F., Núñez, D., Arismendi, I., 2007. “Estudio preliminar de Lineamientos Específicos para la Generación de un Plan de Desarrollo de la Pesca Recreativa de Panguipulli”. SERCOTEC, Valdivia-Chile.
49. Muñoz, C., Jara, A., Beltrán, M., Zúñiga, A., Victoriano, P., Habit, E., 2010. Distribución de la familia Diplomystidae (pisces: siluriformes) en Chile: nuevos registros. Boletín de Biodiversidad de Chile 4, 6-17.
50. Murillo, V., Ruiz, V., 2002. El Puye *Galaxias globiceps* Eigenmann 1927 (Osteichthyes: Galaxiidae): ¿una especie en peligro de extinción?. Gayana 66(2), 191-197. DOI: 10.4067/S0717-65382002000200013.
51. Ortiz-Sandoval, J., Ortiz, N., Cifuentes, R., González, J., Habit, E., 2009. Respuesta de la comunidad de peces al dragado de ríos costeros de la región del BioBío (Chile). Gayana 73(1), 64-75. DOI: 10.4067/S0717-65382009000100010.
52. Ortiz-Sandoval, J., Ortiz, N., Cifuentes, R., González, J., Habit, E., Palma, A., González, J., Reyes, C., Ramos, R., 2013. Biodiversidad y estructura comunitaria de ríos en las zonas árida, semiárida y mediterránea-norte de Chile. Revista Chilena de Historia Natural. 86,1–14. DOI: 10.4067/S0716-078X2013000100001.
53. Palma, A., González, J., Reyes, C., Ramos, R., 2013. Biodiversidad y estructura comunitaria de ríos en las zonas árida, semiárida y mediterránea-norte de Chile. Revista Chilena de Historia Natural 86, 1–14. DOI: 10.4067/S0717-65382009000100010.
54. Parenti, L.R., 1984. A taxonomic revision of the Andean killifish genus *Orestias* (Cyprinodontiformes, Cyprinodontidaae). Bull Am Museum Nat Hist. 178, 107–214.
55. Parra, O., Valdovinos, C., Urrutia, R., Cisternas, M., Habit, E., Mardones. M., 2003. Caracterización y tendencias tróficas de cinco lagos costeros de Chile. Limnética 22, 51–83.
56. Peredo, M., Martínez, F., Garófano, V., Atenas, M., Riestra, F., 2009. Base de datos eco-hidrológica de los una herramienta de gestión para los ecosistemas acuáticos ríos de Chile. Gayana 73: 119-129.
57. Quezada-Romegialli, C., Vila, I., Véliz, D., 2009. Nueva especie íctica invasora en aguas continentales de Chile central: *Jenynsia multidentata* (Jenyns, 1842) (Cyprinodontiformes: Anablepidae). Gayana 73, 74–77. DOI: 10.4067/S0717-65382009000200007.
58. Rosenfeld, C., 2008. Informe final: “Determinación de prevalencia de los virus de la Anemia Infecciosa del Salmón (ISA) y de la Enfermedad Pancreática (PD), en la población de peces silvestres y asilvestrados de la Región de La Araucanía, De Los Ríos, De Los Lagos, Aysén y Magallanes con concesiones de salmonicultura”. Universidad Austral De Chile.
59. Ruiz, V.H., 1993. Ictiofauna del río Andalién (Concepción, Chile). Gayana Zoología 57(2), 109-278.
60. Ruiz, V.H., López, M.T., Moyano H.I., Marchant, M., 1993. Ictiología del alto Biobío: Aspectos taxonómicos, alimentarios, reproductivos y ecológicos con una discusión sobre la hoya. Gayana Zoología 57, 77-88.
61. Ruiz, V.H., Marchant, M., 2004. Ictiofauna de Aguas Continentales Chilenas” Universidad de Concepción. Departamento de Zoología.
62. Salas, D., Véliz, D., Scott, S., 2012. Diferenciación morfológica en especies del género *Cheirodon* (Ostariophysi: Characidae) mediante morfometría tradicional y geométrica. Gayana 76, 142-152. DOI: 10.4067/S0717-65382012000300007.
63. Sepúlveda, M., Arismendi, I., Soto, D., Jara, F., Farias, F., 2013. Escaped farmed salmon and trout in Chile: incidence, impacts, and the need for an ecosystem view. Aquacult. Environ Interact. 4, 273–283.
64. Servicio Nacional de Pesca y Acuicultura, 2012. Fichas Ícticas de Especies en Chile Pesca Recreativa: Especies Dulceacuícolas Protegidas. Ministerio de Economía Fomento y Turismo.
65. Servicio Nacional de Pesca y Acuicultura, 2012. Guía de pesca recreativa: Región de Aysén. Ministerio de Economía. Fomento y Turismo. Gobierno de Chile.
66. Soto, D., Vega, R., Vila, I., 1998. Categorías de conservación de peces nativos de aguas continentales de Chile. Boletín del Museo Nacional de Historia Natural 46: 101-122.
67. Soto, D., Arismendi, I., 2005. Fauna íctica de la cuenca del río Bueno: relevancia de los afluentes en la conservación de especies nativas. Historia, Ecología y biodiversidad de los bosques de la cordillera de la costa de Chile. C. Smith-Ramírez, J. Armesto & C. Valdovinos Eds. Editorial Universitaria 418-426.
68. Soto, D., Arismendi, I., González, J., Sanzana, J., Jara, F., Jara, C., Guzman, E., Lara, A., 2006. Southern Chile, trout and salmon country: invasion patterns and threats for native species. Revista Chilena de Historia Natural. DOI: 10.4067/S0716-078X2006000100009.
69. Soto, M.Á., Castro, J., Walker, L., Malabra, L., Santos, M., Almeida, M., Moreira, O., Artoni, R., 2018. Evolution of trans-Andean endemic fishes of the genus *Cheirodon*(Teleostei: Characidae) are associated with chromosomal rearrangements. Revista chilena de historia natural, 91, 8.91: 8. Doi: 10.1186/s40693-018-0078-5.
70. Unmack, P., Bennin, A., Habit, E., Victoriano, P., Johnson, J., 2009. Impact of ocean barries, topography, and glaciation on the phylogeography of catfish *Trichomycterus areolatus* (Teleostei: Trichomycteridae) in Chiles. Biological Journal of the Linnean Society. 97, 876-892. DOI: 10.1111/j.1095-8312.2009.01224.x.
71. Unmack, P., Habit, E., Johnson, J., 2009. Nuevos registros de *Hatcheria macraei* (siluriformes, trichomycteridae) en la provincia chilena. Gayana 73, 102-110. DOI: 10.4067/S0717-65382009000100013.
72. Valdebenito, I., Fletcher, C., Vera, V., Fernández, J., 2009. Factores fisicoquímicos que regulan la motilidad espermática en peces: aspectos básicos y aplicados. Arch. med. Vet. 41, 97-106. DOI: 10.4067/S0301-732X2009000200002.
73. Valdovinos, C., Urrutia, R., Cisternas, M., Habit, E., Mardones, M., 2003. Caracterización y tendencias tróficas de cinco lagos costeros de Chile. Limnética 22, 51–83.
74. Vargas, C., Contreras, M., Vila, I., 2002. Edad y crecimiento de *Odontesthes brevianalis* (günther 1880) en la laguna Conchali (31°53'; 71°32'). Gayana 66(2), 199-202. DOI: 10.4067/S0717-65382002000200014.
75. Véliz, D., Catalán, L., Pardo, R., Acuña, P., Díaz, A., Poulin, E., Vila1, I., 2012. El género *Basilichthys* (Teleostei: Atherinopsidae) analizado a lo largo de su distribución en Chile (21° a 40° S), utilizando rasgos morfológicos y variabilidad del ADN mitocondrial. Revista Chilena de Historia Natural 85, 49-59. DOI: 10.4067/S0716-078X2012000100004.
76. Vieta, E., 2010. Memoria de Título: Caracterización de los peces en cinco lagos del sur de Chile. UACH. Valdivia.
77. Vila. I., Pinto, M., 1986 A new species of killifish (Pisces, Cyprinodontidae from the Chilean Altiplano. Revue Hydrobiologie Tropical 19 (3-4): 233-239.
78. Vila, I., Fuentes, L., Contreras, M., 1999. Peces límnicos de Chile. Boletín del Museo Nacional de Historia Natural 48, 61-75.
79. Vila, I., Pardo, R., 2006. Peces Límnicos en Biodiversidad de Chile Patrimonio y Desafíos. Comisión Nacional del Medio Ambiente 306-311.
80. Vila, I., Pardo, R., Dyer, B., Habit, E., 2006. Peces Límnicos: Diversidad, origen y estado de conservación en Macrófitas y vertebrados de los sistemas límnicos de Chile. I. Vila, A. Veloso, R. Schlatter & C. Ramírez Eds.
81. Vila, I., Scott, S., Lam, N., Iturra, P., Méndez, M.A., 2010. Karyological and morphological analysis of divergence among species of the killifish genus *Orestias* (Teleostei: Cyprinodontidae) from the southern Altiplano. In: Nelson JS, Schultze H-P, Wilson MVH, editors. Origin and Phylogenetic Interrelationships of Teleosts. München: Verlag Dr. Friedrich Pfeil 471–480.
82. Vila, I., Habit, E., 2015. Current situation of the fish fauna in the Mediterranean region of Andean river systems in Chile. Fishes in Mediterranean Environmentes 2015.002,19 p. DOI: 10.29094/FiSHMED.2015.002.
83. Vila, I., Quezada-Romegialli, C. 2018. Peces límnicos de Chile. Importancia biogeográfica y ecológica, conocimiento actual y vacíos taxonómicos. In: Biodiversidad de Chile, Patrimonio y Desafíos, 3a. Ed. Ministerio Del Medio Ambiente. Santiago De Chile: In press.
84. Zunino, S., Aliaga, C., Da Venezia, P., 2009. Comunidades de peces en desembocaduras de ríos y esteros de la Región de Valparaíso, Chile central. Revista de biología marina y oceanografía 44(1), 123-130. DOI: 10.4067/S0718-19572009000100011.
